# Regulation of Cx43 Gap Junction Intercellular Communication by Bruton’s Tyrosine Kinase and Interleukin-2-Inducible T-Cell Kinase

**DOI:** 10.3390/biom13040660

**Published:** 2023-04-08

**Authors:** Ishika Basu, Hanjun Li, Andrew J. Trease, Paul L. Sorgen

**Affiliations:** Department of Biochemistry and Molecular Biology, University of Nebraska Medical Center, Omaha, NE 68198, USA

**Keywords:** Cx43, gap junctions, phosphorylation, BTK, ITK

## Abstract

T and B cell receptor signaling involves the activation of Akt, MAPKs, and PKC as well as an increase in intracellular Ca^2+^ and calmodulin activation. While these coordinate the rapid turnover of gap junctions, also implicated in this process is Src, which is not activated as part of T and B cell receptor signaling. An in vitro kinase screen identified that Bruton’s tyrosine kinase (BTK) and interleukin-2-inducible T-cell kinase (ITK) phosphorylate Cx43. Mass spectroscopy revealed that BTK and ITK phosphorylate Cx43 residues Y247, Y265, and Y313, which are identical to the residues phosphorylated by Src. Overexpression of BTK or ITK in the HEK-293T cells led to increased Cx43 tyrosine phosphorylation as well as decreased gap junction intercellular communication (GJIC) and Cx43 membrane localization. In the lymphocytes, activation of the B cell receptor (Daudi cells) or T cell receptor (Jurkat cells) increased the BTK and ITK activity, respectively. While this led to increased tyrosine phosphorylation of Cx43 and decreased GJIC, the cellular localization of Cx43 changed little. We have previously identified that Pyk2 and Tyk2 also phosphorylate Cx43 at residues Y247, Y265, and Y313 with a similar cellular fate to that of Src. With phosphorylation critical to Cx43 assembly and turnover, and kinase expression varying between different cell types, there would be a need for different kinases to achieve the same regulation of Cx43. The work presented herein suggests that in the immune system, ITK and BTK have the capacity for the tyrosine phosphorylation of Cx43 to alter the gap junction function in a similar manner as Pyk2, Tyk2, and Src.

## 1. Introduction

Gap junctions provide for direct cell-to-cell communication to exchange ions and small metabolites [1]. They are formed from two connexons, one from each cell, that are each comprised of six connexins. Connexin43 (Cx43), the most widely studied connexin isoform, is extensively regulated by protein partner interactions and multiple phosphorylation events in the carboxyl terminal domain (CT) [2]. For example, microtubules and the actin cytoskeleton are necessary for Cx43 trafficking from the trans-Golgi network to the plasma membrane [3,4,5,6,7]. ZO-1 (binds to actin) regulates the size of the gap junction plaques by controlling the rate of channel accretion at the plaque perimeters [8]. Ezrin (binds to actin) binds Cx43 to promote PKA-mediated phosphorylation, which enhances gap junction assembly and communication [9,10]. At the plaque, Cx43 interacts with both microtubules and Drebrin (binds to actin) to maintain gap junctions in their functional state [4,11]. Cx43 has a short half-life of only 1–5 h [12,13]. Rapid internalization may be an important mechanism for adjusting intercellular coupling under normal (e.g., cell migration, mitosis) and pathophysiological (e.g., ischemia, hearing loss, oculodentodigital dysplasia) conditions [14,15]. Internalization from the plasma membrane can occur by the disaggregation of connexons into their parent cell, or as vesicle-like double-membrane structures termed annular gap junctions or connexosomes [16,17,18]. Cx43 can be degraded by proteasomal, lysosomal, and autophagosomal mechanisms (for review, see [19,20,21]).

Src phosphorylation and Nedd4 ubiquitination of Cx43 are separate events that trigger gap junction closure, internalization, and degradation [22,23,24,25,26,27,28,29,30,31,32,33]. One impact of Src phosphorylation on channel activity is to decrease electrical coupling by reducing the open probability and changes in selectivity [34]. Another consequence of Src phosphorylation that leads to a decrease in gap junctional intercellular communication is inhibiting protein partners that stabilize Cx43 at the plaque. For example, Src phosphorylation of Y247 inhibits the Cx43 interaction with β-tubulin, and Y265 and Y313 inhibit the interaction with Drebrin [24,35,36]. Src also directly interacts with ZO-1, causing ZO-1 displacement from the Cx43CT [37]. This initially causes the plaque size to increase and then be removed from the plasma membrane as it is no longer anchored to the cytoskeletal network (also caused by the loss of interaction with tubulin and Drebrin) [8,38]. Of note, the Cx43CT domain contains two tyrosine sorting motifs (Y265-F268, and Y286-V289) for the interaction with AP2 to initiate clathrin-mediated internalization [39]; the first of which is a Src phosphorylation site. Based upon the premise that phosphorylation of Y265 would inhibit the binding of AP2 [40] and mutation of Y265 partially impairs internalization [39], Src phosphorylation can alter the kinetics of internalization or potentially trigger turnover via the proteosome or other pathways leading to the lysosome (e.g., clathrin-independent mechanisms) [39,41,42,43]. The complexity of Cx43 internalization is further complicated in that the binding sites for AP2 and Nedd4 overlap [31,39]. Phosphorylation of Cx43 residues S279 and S282 by MAPK increases the binding affinity for Nedd4, which leads to ubiquitination, Eps15 recruitment, and internalization [21,31,33,41,44]. The mechanism for Cx43 internalization when the AP2 binding sites are disrupted is via connexosome (or annular gap junctions) formation [43,45]. Evidence suggests that the degradation of connexosomes occurs though lysosomal degradation pathways, the phago-/lysosomal or the endo-/lysosomal (for review, see [19,46]).

With the phosphorylation of Y247, Y265, and Y313 playing such an integral role in Cx43 regulation as well as proteomic discovery-mode mass spectrometry (MS) data identifying other tyrosine residues (Y267, Y286, and Y301) as potential phosphorylation targets (PhosphoSitePlus web site), we performed an in vitro kinase screen (Eurofins Scientific’s KinaseProfiler) to identify whether other tyrosine kinases phosphorylate Cx43. The screen identified tyrosine kinase 2 (Tyk2) and protein tyrosine kinase 2 beta (Pyk2), both of which function to decrease Cx43 gap junction intercellular communication in a manner similar, but different than Src [47,48]. The phosphorylation of Cx43 by Tyk2 was Src-independent and angiotensin II activation of Tyk2 increased the intracellular protein level of Cx43 via STAT3 [47]. The phosphorylation of Cx43 by Pyk2 was Src-dependent and the inhibition of Pyk2 alone completely reversed the effect of PMA (phorbol-12-myristate-13-acetate) on neonatal rat ventricular myocytes [48]. Additionally, an animal model of myocardial infarction-induced heart failure showed a higher level of active Pyk2 activity and increased interaction with Cx43 in ventricular myocytes at the intercalated disc and lateral membrane [48]. As part of the screen, Bruton’s tyrosine (BTK) and interleukin-2-inducible T-cell (ITK) kinases were also identified to phosphorylate the purified Cx43CT domain [49,50]. ITK and BTK are members of the Tec family of kinases, which are primarily expressed in cells of hematopoietic lineage [51]. Based upon the following correlations: (1) Cx43-mediated signaling occurs at the immunological synapse (and non-synaptical regions) [52,53], (2) BTK and ITK are activated via the B-cell and T-cell receptors at the immunological synapse [54,55], (3) down-stream signaling cascades upon B-cell and T-cell receptor activation do not directly involve activation of c-Src [54,56], and (4) phosphorylation of Cx43 residues Y247 and Y265 is necessary for B-cell spreading in response to B-cell receptor signaling [57], we tested whether the activation of BTK and ITK regulate Cx43 in a similar manner as Pyk2, Tyk2, and Src.

## 2. Materials and Methods

### 2.1. Antibodies and Detection Reagents

α-Vinculin (#4650S, Western blot dilution [WB]—1:1000), α-BTK (#8547S, WB—1:1000, Immunofluorescence [IMF]—1:200), α-phospho-BTK (#5082S, WB—1:1000, IMF—1:100), α-ITK (#2380S, WB—1:1000, IMF—1:200), α-rabbit-Alexa 488 (#4413, IMF—1:500), α-mouse-Alexa647 (#4410, IMF—1:500), anti-rabbit IgG (#7074S, WB—1:5000), and anti-mouse IgG (#7076S, WB—1:5000) were purchased from Cell Signaling (Danvers, MA, USA); α-phospho-ITK (#PA5-40292, WB—1:1000, IMF—1:100) was purchased from Invitrogen (Waltham, MA, USA); α-Cx43 (#C6219, WB—1:2000, IMF—1:500) and α-Actin (#A5441, WB—1:5000) were purchased from Sigma (Burlington, VT, USA); F(ab’)2-Goat anti-human IgM (referred hereafter as α-IgM, see Section 2.6 for concentration) was purchased from Invitrogen (Waltham, MA, USA) (#A24484); Lucifer Yellow (#L453, 2.5 mg/mL, was purchased from Life Technologies, Carlsbad, CA, USA); DAPI (#5748) was purchased from Tocris (Bristol, United Kingdom, for staining concentration, see Section 2.10). Additionally, α-CD3 (clone OKT3) and α-CD28 antibodies were purchased from BioLegend (San Diego, CA, USA, #317317) and Invitrogen (Waltham, MA, USA, #16-0289-81), respectively (see Section 2.6 for concentration). The Cx43 pY247 antibody was custom made from LifeTein (Somerset, MA, USA) and used in a previous publication [48]. This antibody was validated in HeLa cells ± Cx43 expression, in HEK-293T ± Pyk2 expression, and with peptides ± phosphorylation at Y247.

### 2.2. Kinase Screen

Purification of rat Cx43CT (residues V236-I382) was conducted as previously described [58,59]. The purified protein was sent to Eurofins Scientific (Dundee, UK) to perform an in vitro kinase screening assay. Cx43CT was phosphorylated by the catalytic domains of BTK and ITK. Each kinase had a positive control peptide for comparison. The in vitro phosphorylation reaction was incubated at 30 °C for 40 min and then transferred to P30 Filtermat for substrate capture. The Merck Millipore radiometric assay was used to determine the phosphorylation levels.

### 2.3. Mass Spectrometry

Purified Cx43CT was incubated with active BTK or active ITK overnight at 30 °C. After stopping this reaction with ice, 10 nmol of protein was run on an SDS-PAGE gel followed by Coomassie Blue staining. The Cx43CT bands were excised and sent to the Harvard Center for Mass Spectrometry.

### 2.4. Cell Culture

HEK-293T cells (gift from Dr. Myron Toews, University of Nebraska Medical Center, Omaha, VT, USA) were cultured in Dulbecco’s modified Eagle medium (DMEM; 10% fetal bovine serum, 2 mM L-glutamine, 1% pen-strep, and 0.2% normocin) were added at 37 °C in a 5% CO_2_ atmosphere. Puromycin (2 µg/mL) was used as a selective antibiotic when required. Cells were passaged after reaching 95% confluency. Daudi (#CCL-213) and Jurkat (TIB-152) cells were purchased from ATCC and cultured in RPMI 1640 medium at 37 °C in a humidified 5% CO_2_ atmosphere. The RPMI 1640 solution was supplemented with 25 mM D-glucose, 18 mM sodium bicarbonate, 10 mM HEPES, 1 mM sodium pyruvate, 10% fetal bovine serum, and 1% pen-strep (pH 7.4). Passaging of cells (at 1 × 10^5^ cells/mL) was carried out by centrifugation at 126 × *g*, 8 min, and resuspending the pellet in fresh media or by directly adding cells (at 1 × 10^5^ cells/mL) to fresh media at 20% volume.

### 2.5. Stable Clone Generation

The BTK or ITK constructs were cloned into the expression vector pD2529 using the NEBuilder HiFi DNA Assembly technology (New England BioLabs, Ipswich, MA, USA). *E.coli* strain DH5α was transformed with the cloned plasmid. Midiprep of the plasmids was conducted with a Qiagen Kit. The 60% confluent HEK-293T cells were transfected with BTK or ITK plasmids using the lipofectamine reagent in Opti-MEM and antibiotic free DMEM. Stable cells were selected using 2 µg/mL puromycin. Clones were screened using Western blot and immunofluorescence. The clones chosen for further investigation were based upon protein expression and cellular localization.

### 2.6. Activation of Endogenous BTK and ITK

Daudi (B lymphocyte isolated from the peripheral blood of a 16-year-old male, Burkitt’s lymphoma patient) cells were seeded to reach 1 × 10^7^ cells/mL density. Then, 10 µg/mL of the α-IgM antibody was added to the cells and incubated at 37 °C for 45 min (optimized and modified from [60]). The cells were then lysed prior to Western blot analysis. Jurkat (T lymphocyte cell line established from the peripheral blood of a 14-year-old, male, acute T-cell leukemia patient) cells were seeded to until 1 × 10^7^ cells/mL density. Next, 3 µg/mL of the α-CD 3 antibody was added to the plates to coat it and stored overnight at 4 °C, followed by the addition of the α-CD 28 antibody (5 µg/mL), and incubated for 2 h at 37 °C before lysing the cells for Western blot (optimized and modified from [61,62]).

### 2.7. GST Pull-Down Assay

Recombinantly expressed and purified GST only protein and GST-Cx43CT (V236-I382) fusion proteins were attached to glutathione beads in a buffer with 50 mM HEPES (pH 7.4), 150 mM NaCl, 1 mM DTT, 0.5% Triton X-100, and Roche cOmplete protease inhibitor [58,59]. Daudi or Jurkat cell lysates (1 mL normalized to 2 mg/mL) were incubated with GST control, GST-Cx43CT or GST-Cx43CT, and DTSSP (2 mM final concentration) for 6 h at 4 °C. Elution of bound proteins was carried out with SDS-PAGE sample buffer (69% 4× Tris-Cl/SDS, 29% glycerol, 0.3 M SDS, 0.6 M DTT, 0.19 mM bromophenol blue, dissolved in water, pH 6.8), boiled for 5 min at 95 °C, and analyzed by Western blot.

### 2.8. Western Blot

Confluent cells were washed in 1× TBS and lysed in a buffer containing protease and phosphatase inhibitors, 2 mM PMSF, 2 mM Pepstatin A, and 1% Triton X-100. The bicinchoninic acid assay (BCA) was used to measure the concentration of lysates. Then, 6× loading dye (69% 4× Tris-Cl/SDS, 29% glycerol, 0.3 M SDS, 0.6 M DTT, 0.19 mM bromophenol blue, dissolved in water, pH 6.8) was added to the lysate without boiling and 30 µg of protein was loaded on a 10% SDS-PAGE gel, followed by transfer to the PVDF (Millipore) membrane. Blocking of the membrane was conducted in 5% bovine serum albumin (BSA)/TBS with Tween-20 for 1 h before primary antibody incubation overnight. The membrane was washed in TBS with Tween before secondary antibody incubation for 1 h. Signal detection was conducted with the SuperSignal West Femto (Thermo Scientific, Waltham, MA, USA) substrate using a digital imager (iBright, Thermo Fisher Scientific). Quantification was the result of measuring the sum of intensity of all bands shown in the blot.

### 2.9. Triton X-100 Solubility Assay

Fully confluent HEK-293T cells were lysed in 1× PBS containing Roche cOmplete protease inhibitor and sonicated once for 10 s. Lysate concentration was determined by BCA and normalized. A sample of the lysate was reserved and mixed with 6× loading dye to be used for the total protein fraction (T). The rest of the lysate was subjected to 1% TX-100 solubilization by 1 h agitation at 4 °C. This was further fractionated into soluble (S, non-gap junctional) and insoluble (I, gap-junctional) fractions by ultracentrifugation (100,000 × G, 1 h, 4 °C). The insoluble fraction was solubilized in a buffer containing 1× PBS, 8 M urea, 2.5% SDS, 0.1 M DTT, 1× Roche + EDTA, PMSF, and Pepstatin A. The total (T), soluble (S), and insoluble (I) fractions were analyzed by Western blot after mixing with 6× loading dye.

### 2.10. Immunofluorescence

Glass coverslips were coated with fibronectin (at 1:50 dilution in 1× PBS) for 2 h and cells were seeded onto them. Coverslips with confluent cells were washed gently with 1× PBS and fixed with 3.7% formaldehyde in 1× PBS at room temperature for 20 min. They were then incubated with blocking buffer (1% BSA and 0.3% Triton X-100) at room temperature for 1 h. Primary antibodies were added and incubated overnight at 4 °C. Coverslips were then washed with 1× PBS before incubation with the secondary antibodies for 1 h at room temperature. Then, 100 ng/mL DAPI was added and washed prior to mounting with SlowFade anti-fade (Life Tech). Cells were imaged by confocal microscopy. For the HEK-293T cells (parental and stable clones), the plaque area was quantified. The area of each plaque (*n* = 10 for each replicate) was measured separately in pixels using the ImageJ application (1.53 t) and the average area value per plaque was calculated, which is indicated on the *y*-axis. The statistical data were generated on GraphPad Prism 9.0.0 using the Student’s *t*-test.

### 2.11. Confocal Imaging

All cell immunofluorescence images were acquired on a Zeiss LSM 800 Confocal System (Jena, Germany) using appropriate numerical aperture objectives and appropriate filter sets.

### 2.12. Dye Transfer Assay

Scrape loading of the HEK-293T cells was conducted as described previously [48]. All reagents were maintained at 37 °C during the experiment. Cells were seeded on plates coated with bovine plasma fibronectin (at 1:50 dilution in 1× PBS for 2 h). Confluent cells were gently washed with 1× PBS and dye containing Lucifer Yellow (2.5 mg/mL) and Texas Red Dextran (1 mg/mL), dissolved in 1× PBS, was added. Texas Red Dextran was used as a control for the scrape as the molecular weight was too large to pass through a gap junction (3 kDa; data not shown). The cells were scraped with a scalpel and incubated with the dye at room temperature for 5 min. The dye was removed before washing the cells with 1 mM CaCl_2_ and 1 mM MgCl_2_ in 1× PBS. The cells were incubated with antibiotic-free DMEM for 5 min at 37 °C. Then, 4% PFA was used to fix the cells for 20 min at room temperature, followed by DAPI (in 1× TBST) staining for another 20 min at room temperature and mounting (with 25% antifade, 75% glycerol) before imaging with a confocal microscope. We measured the total surface area of the dye transferred on both sides of the scrape using ImageJ. During quantification, the surface area of the dye transferred in the HEK^Par^ cells was normalized to 1. Dye transfer in HEK^BTK^ and HEK^ITK^ are shown as the fraction of dye transfer compared to HEK^Par^. Statistical data were generated using the ImageJ application (1.53t) and GraphPad Prism (units were measured in pixels).

### 2.13. Parachute Assay

Plates of donor and recipient cells were grown to confluency. The donor plate was incubated with the cell-permeant dyes Dil (#42364, Sigma-Aldrich, Burlington, VT, USA) and calcein AM (#14948, Cayman Chemical Company, Ann Arbor, MI, USA) for 20 min at 5 µM and 1 µM, respectively. Dil is a carbocyanine dye that is confined to the membrane cell. The nonfluorescent calcein AM is a cell-permeant dye that is converted to a green fluorescent calcein after acetoxymethyl ester hydrolysis by intracellular esterases. Calcein can only pass-through gap junctions [63,64]. Donor cells (one donor plate was used for both untreated and treated samples for each experiment) were then added to recipient cells at 1:100 dilution and incubated at 37 °C for the indicated time points. After 240 min of incubation, the activating antibodies were added, and the cells were further incubated for the time needed by the kinases to become activated (see Section 2.6). This was followed by confocal imaging to determine whether the green fluorescent dye from the donor cells was transferred into the recipient cells. Such transfer is a positive indication of gap junction intercellular communication. At least 10 random microscopic fields per group were photographed and counted, and the experiments were repeated three times. Statistical analysis was done by GraphPad Prism. *Y*-axis units were calculated as the number of recipient cells per donor cell.

### 2.14. Statistical Analysis

All data were analyzed by using GraphPad Prism 8.0 and presented as the mean ± SD. Statistical analysis performed in GraphPad Prism 8.0 were either one-way ANOVA with a Neuman-Keuls post hoc analysis or Student’s *t*-test where appropriate. *p*-values < 0.05 were considered statistically significant.

## 3. Results

### 3.1. BTK and ITK Directly Interact with and Phosphorylate the Cx43CT Domain

**Figure 1:**
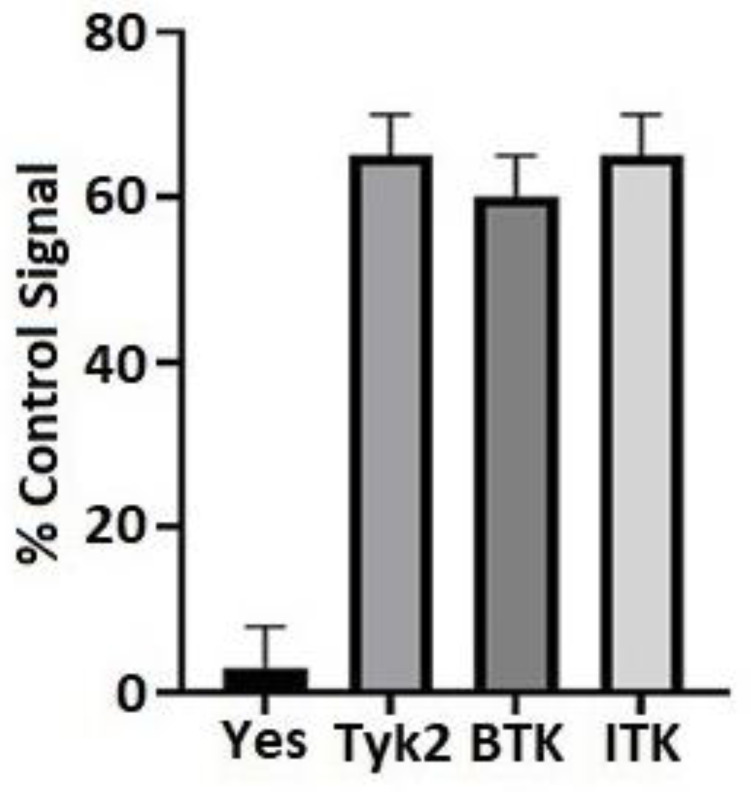
Identifying novel kinases that phosphorylate Cx43. In vitro kinase screening was performed using purified Cx43CT and the catalytic domains of BTK and ITK. A positive control peptide was used for each kinase (100% signal) to calculate the Cx43CT phosphorylation signal. The Src and Jak family members Yes and Tyk2 were shown to illustrate negative and positive results from the screen, respectively.

**Figure 2:**
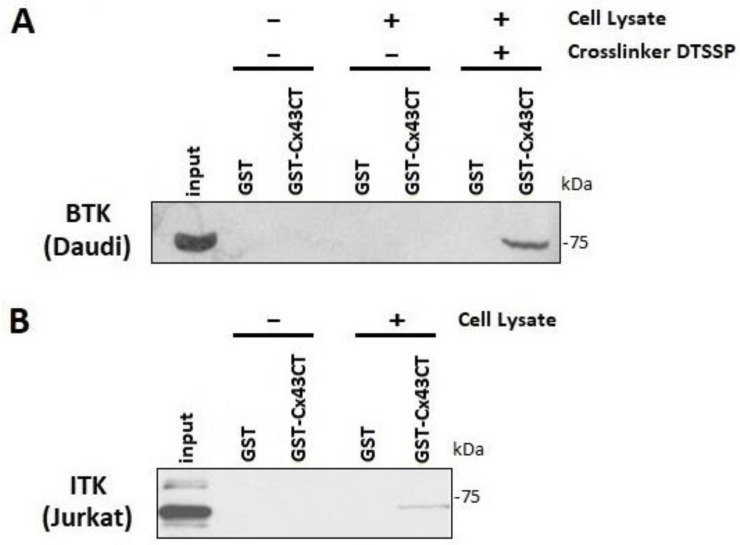
Confirming the direct Cx43 interaction with BTK and ITK. (**A**) GST pull-down assay was performed in Daudi cells with GST (control), GST-Cx43CT immobilized on glutathione beads, and GST-Cx43CT in the presence of crosslinker DTSSP. Pulled-down proteins were analyzed by Western blot using an anti-BTK antibody. (**B**) The GST pull-down assay was conducted in Jurkat cells with GST only and GST-Cx43CT immobilized on glutathione beads. Pulled-down proteins were analyzed by Western blot using an anti-ITK antibody.

An in vitro tyrosine phosphorylation screen performed by Eurofins Scientific (KinaseProfiler, Dundee, UK) found that BTK and ITK phosphorylated purified Cx43CT_V236-I382_ (Figure 1). To confirm the interaction of BTK and ITK with Cx43, purified GST-tagged Cx43CT_V236-I382_ was immobilized on glutathione-Sepharose beads and lysate from Daudi cells that express BTK and Jurkat cells that express ITK were used in a pull-down assay (Figure 2). Multiple attempts were unsuccessful in the GST-Cx43CT pull-down of BTK. However, the BTK interaction with the Cx43CT was stabilized by the chemical cross-linker 3,3′-dithiobis (sulfosuccinimidylpropionate) (DTSSP), which can capture weak and/or transient interactions [65] (Figure 2A). The GST-Cx43CT was able to pull-down ITK from the Jurkat cells without the use of DTSSP, suggesting a higher binding affinity than BTK (Figure 2B).

### 3.2. BTK and ITK Phosphorylate Cx43CT Tyrosine Residues

Purified Cx43CT_V236-I382_ was incubated in vitro with active BTK and ITK (Life Technologies) as described in [66]. After trypsin digestion, tandem MS/MS identified phosphorylation for both BTK and ITK at five of the six tyrosine residues (Y247, Y265, Y267, Y286, and Y313), however, the greatest number of detected phospho-peptides belonged to residues Y247, Y265, and Y313 (Table 1 and Table 2). To determine whether the BTK and ITK phosphorylation of Cx43 occurred in cells, a HEK-293T cell line (Cx43, endogenously expressed) was created to stably express either BTK or ITK (HEK^BTK^ and HEK^ITK^).

### 3.3. BTK and ITK Phosphorylation of Cx43 Decreases the P2 Isoform and Increases Tyrosine Phosphorylation in HEK Cells

**Figure 3:**
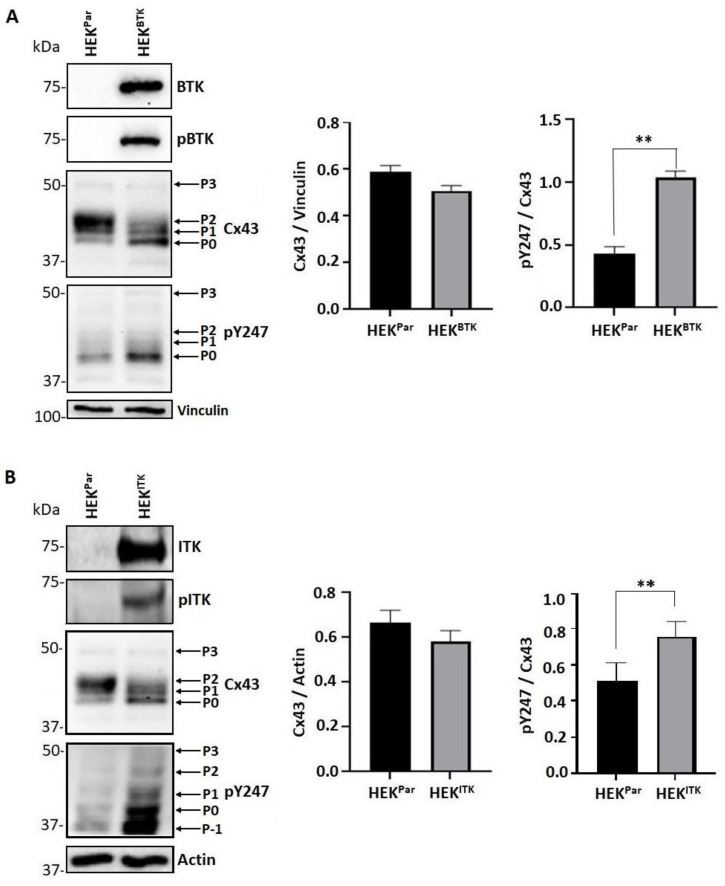
Effect of BTK and ITK phosphorylation on Cx43 regulation in the HEK-293T cells as observed through changes in electrophoretic mobility. Western blot of HEK-293T parental (HEK^Par^) and (**A**) stably expressing BTK (HEK^BTK^) or (**B**) stably expressing ITK (HEK^ITK^) cell lysates. Antibodies used are labeled on the right of each blot. Arrows are pointing to the different migrating Cx43 bands from the electrophoresis. Data were quantified using ImageJ (all units were measured in pixels) and GraphPad Prism by the Student’s *t*-test (*n* = 3, ** *p* < 0.01).

For both the HEK^BTK^ and HEK^ITK^ cell lines, the kinases were observed to be in their active state (pBTK and pITK) and there was no statistical change in the level of total Cx43 protein compared to HEK-293T (HEK^Par^; Figure 3A,B). For BTK and ITK, the presence of the active kinase caused a change in the migration pattern of Cx43 in the SDS-PAGE gel from the P2 isoform to P1 and P0 (predominately) isoforms. The change in electrophoretic mobility was in tandem with an increase in phosphorylation at the Cx43 residue Y247. The effect of ITK phosphorylation on Cx43 was somewhat different to that of BTK in that the phosphorylation of Y247 (pY247) was found in an additional slower band (P-1, potentially a truncated Cx43 isoform) and multiple faster-migrating bands.

### 3.4. BTK and ITK Phosphorylation Decreases the Cx43 Plaque Area in HEK-293T Cells

**Figure 4:**
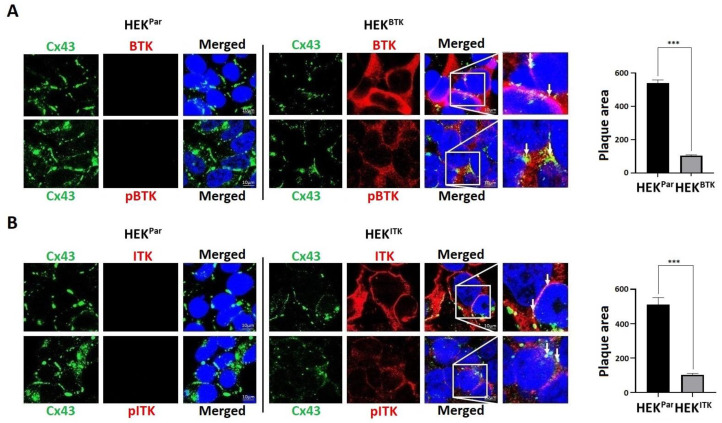
Cellular localization of endogenous Cx43 in the HEK-293T cells overexpressing active BTK or ITK. Immunofluorescence was performed to visualize Cx43 in the HEK-293T cells stably expressing (**A**) BTK (HEK^BTK^) or (**B**) ITK (HEK^ITK^). HEK-293T parental (HEK^Par^) cells were used as the control (Green, Cx43; Red, BTK/pBTK or ITK/pITK; Blue, DAPI; white arrows show colocalization with Cx43). Surface area of the plaques was measured on ImageJ (all units were measured in pixels) and quantified using GraphPad Prism by Student’s *t*-test (*n* = 10, *** *p* < 0.001).

Here, we tested whether Cx43 colocalizes with BTK or ITK. Cx43 localized to both the plasma membrane and intracellular compartments in the HEK^Par^ (Figure 4A,B). The HEK^Par^ showed large defined plaques in the areas between cells. These become less pronounced and more diffuse in the presence of either kinase. There was a significant decrease in the Cx43 plaque area in the presence of active BTK and ITK. Both the total and active BTK and ITK were localized all throughout the cell and at the plasma membrane. Even though saturation of the total BTK and ITK kinases would hinder any determination of co-localization, there was some colocalization observed with the active kinases. Based upon the observed Cx43 phosphorylation caused by BTK and ITK, the data suggest that these kinases do not remain attached to Cx43 once phosphorylation has occurred.

### 3.5. BTK and ITK Phosphorylation of Cx43 Decreases Gap Junction Intercellular Communication in HEK-293T Cells

**Figure 5:**
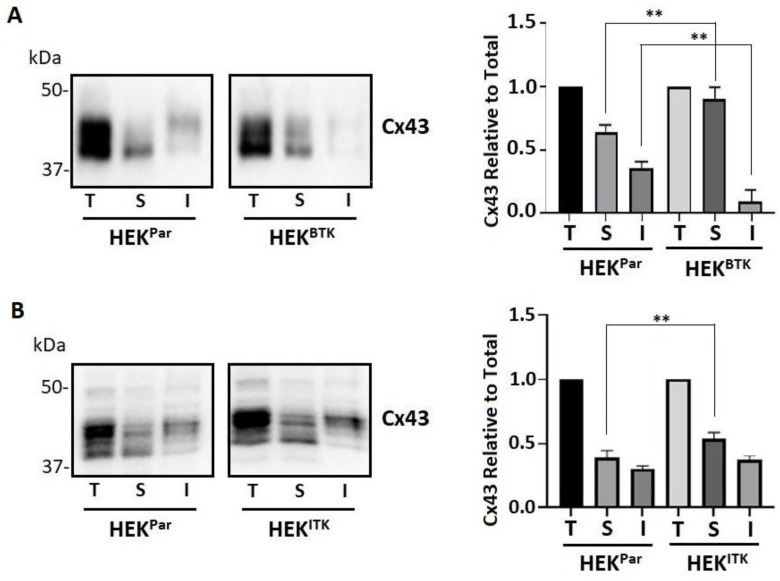
The effect of BTK and ITK phosphorylation on Cx43 regulation in the HEK-293T cells as observed through changes in detergent solubility. Cx43 was extracted with 1% Triton X-100 from HEK-293T parental (HEK^Par^) and (**A**) stably expressing BTK (HEK^BTK^) or (**B**) stably expressing ITK (HEK^ITK^) cells. Equal amounts of the total protein fraction (T), the Triton X-100 soluble fraction (S), and the insoluble fraction (I) were subjected to SDS-PAGE and blotted with an antibody against Cx43. Bands were quantified using ImageJ (all units measured in pixels) and GraphPad Prism. Statistical data represent one-way ANOVA (*n* = 3, ** *p* < 0.01).

To determine whether the increased level of phosphorylation at Y247 caused by BTK or ITK led to a decrease in the amount of Cx43 at the junctional plaque, we assessed the level of Triton X-100 detergent solubility (Figure 5). Cx43 gap junction channels are localized in the detergent-insoluble fraction [67,68]. Conversely, non-communicating membrane localized Cx43 and intracellular Cx43 are found in the soluble fraction [68]. After detergent extraction, Western blot data showed that active BTK (Figure 5A) or ITK (Figure 5B) in the HEK-293T cells increased the detergent-soluble fraction of Cx43. Only for BTK did the increase in the soluble fraction correspond to a statistical decrease in the insoluble fraction. These findings, along with the increase in Y247 phosphorylation and immunofluorescence data, suggest that BTK and ITK contribute to the instability of Cx43 at the junctional plaque.

**Figure 6:**
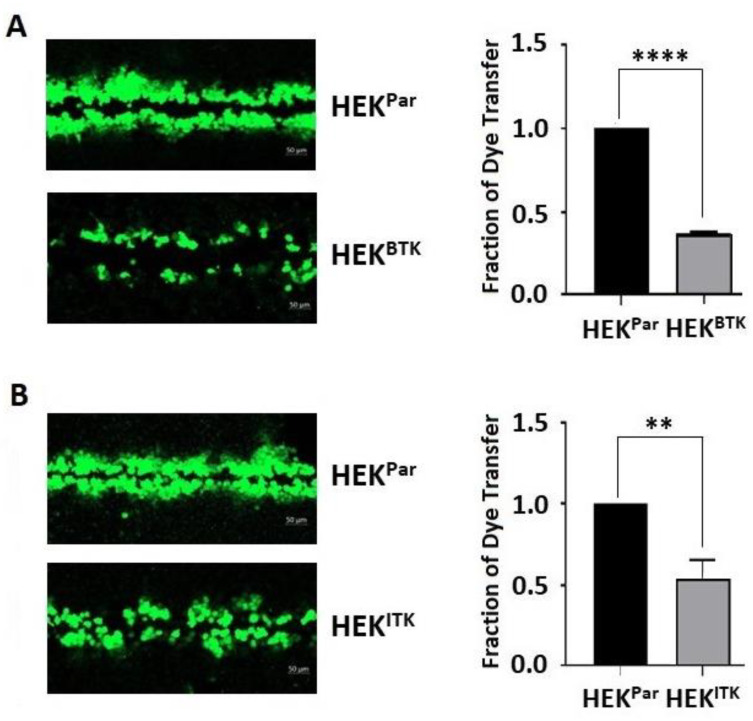
The effect of BTK and ITK phosphorylation on the Cx43 gap junction intercellular communication in HEK-293T cells. A scrape loading dye transfer assay was used to assess communication from HEK-293T parental (HEK^Par^) and (**A**) stably expressing BTK (HEK^BTK^) or (**B**) stably expressing ITK (HEK^ITK^) cells. Representative confocal images (Green, Lucifer yellow) are provided. Statistical data were calculated by ImageJ (all units measured in pixels) and GraphPad Prism by measuring the surface area of the transferred dye compared to HEK^Par^. Data represent the Student’s *t*-test (*n* = 3, ** *p* < 0.01; **** *p* < 0.0001).

To confirm that phosphorylation affects cell-to-cell communication, junctional transfer of the tracer Lucifer Yellow (anionic) was measured in a scrape-loading assay using the HEK^BTK^ and HEK^ITK^ cells compared to HEK^Par^ (Figure 6). Texas Red Dextran (3 kDa; junction impermeable) was used to mark the loaded cells (data not shown). Similar to previously published studies, Cx43 expressing cells like HEK^Par^ were extensively coupled with respect to Lucifer Yellow [24,48]. The presence of active BTK (Figure 6A) and ITK (Figure 6B) reduced the gap junction intercellular communication by ~75% and ~50%, respectively. This observation is consistent with the kinases causing an increase in the soluble pool and phosphorylation of Y247.

### 3.6. Tyrosine Phosphorylation of Cx43 Is Increased after Activation of B and T Lymphoblasts

**Figure 7:**
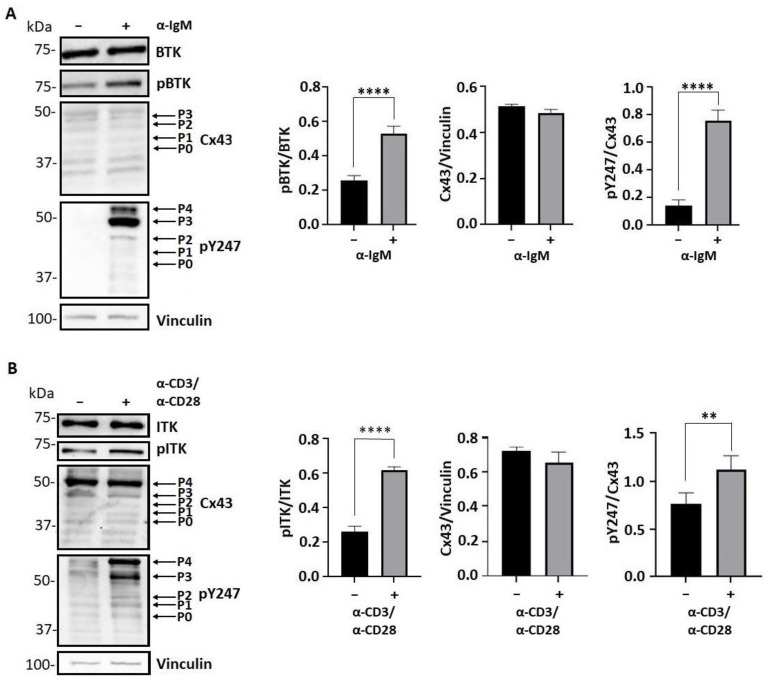
The effect of BTK and ITK phosphorylation on Cx43 regulation in lymphocytes as observed through changes in electrophoretic mobility. Western blot of (**A**) Daudi cells with and without BTK activation using the α-IgM antibody or (**B**) Jurkat cells with and without ITK activation using α-CD3/α-CD28 antibodies. Antibodies used were labeled on the right of each blot. Arrows point to the different migrating Cx43 bands from the electrophoresis. Data were quantified using ImageJ (all units measured in pixels) and GraphPad Prism by Student’s *t*-test (*n* = 3, ** *p* < 0.01; **** *p* < 0.0001).

We next wanted to determine whether the findings observed from using the HEK-293T cells would translate to a more biologically relevant system. Therefore, endogenous BTK and ITK were activated via the B-cell (by α-IgM) and T-cell (α-CD3/α-CD28) receptors in Daudi and Jurkat cells, respectively. Western blot was used to determine whether the activation of BTK and ITK led to an increase in Cx43 tyrosine phosphorylation (Figure 7). There was a significant increase in the level of active BTK (Figure 7A) and ITK (Figure 7B) upon the activation of the B- and T-cell receptors, respectively. Additionally, the activation of BTK and ITK did not affect the Cx43 protein level nor the migration pattern in the SDS-PAGE gel. Interestingly, unlike in the Daudi cells, the total Cx43 in the Jurkat cells had a predominate band at ~50 kDa with or without activation. The near absence of Cx43 in B cells was not unexpected as Cx43 is generally not abundant in B cells (ImmGen database). The strong Cx43 band observed in the Jurkat cells is consistent with bands observed from normal T cells [69]. Activation of the kinases did increase the Cx43 Y247 phosphorylation (pY247). The strongest intensity bands for pY247 were similar between the Daudi and Jurkat cells, with an enrichment in two slower moving bands around ~50 kDa.

### 3.7. Cx43 Co-Localizes with BTK and ITK in Lymphocytes

**Figure 8:**
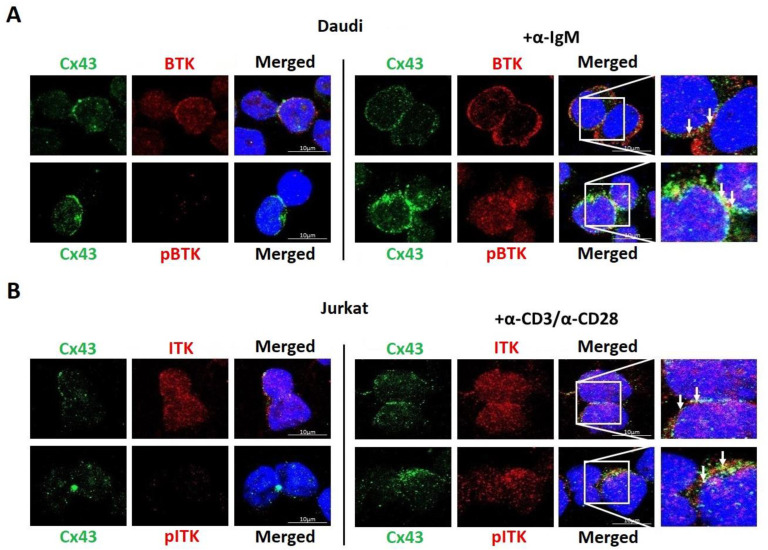
Cellular localization of endogenous Cx43 in lymphocytes with active BTK or ITK. Immunofluorescence was performed to visualize Cx43 in (**A**) Daudi cells with and without BTK activation using the α-IgM antibody or (**B**) Jurkat cells with and without ITK activation using the α-CD3/α-CD28 antibodies (Green, Cx43; Red, BTK/pBTK or ITK/pITK; Blue, DAPI; white arrows show colocalization with Cx43).

Prior to activation, cellular localization of total BTK was diffused throughout the cell, with little-to-no active BTK (Figure 8A). After activation of the B-cell receptor, localization of the total BTK increased at the plasma membrane, and the level of active BTK increased throughout the cell. Cx43 was localized at the plasma membrane and in intracellular compartments with and without BTK activation. There was no change in the level of Cx43 after the activation of BTK. Before the activation of BTK, the entire coating of the plasma membrane by BTK makes it difficult to conclude colocalization with Cx43. However, after activation, there were enriched pockets of active BTK that colocalized with Cx43 at the plasma membrane (yellow color, see white arrows). The findings for BTK in the Daudi cells were like those observed for ITK in the Jurkat cells (Figure 8B). ITK was found throughout the entire cell before and after activation of the T-cell receptor. Cx43 had a similar diffuse localization pattern before and after ITK activation with little-to-no change in the protein level. After activation, there were areas of active ITK that colocalized with Cx43 at the plasma membrane. The one variation between the HEK-293T cells and the lymphocytes upon BTK and ITK activation was the immunofluorescence data. We speculate that a combination of different cell types and level of BTK and ITK activation can attribute to this difference.

### 3.8. Activation of B and T Lymphocytes Decreases Gap Junction Intercellular Communication

**Figure 9:**
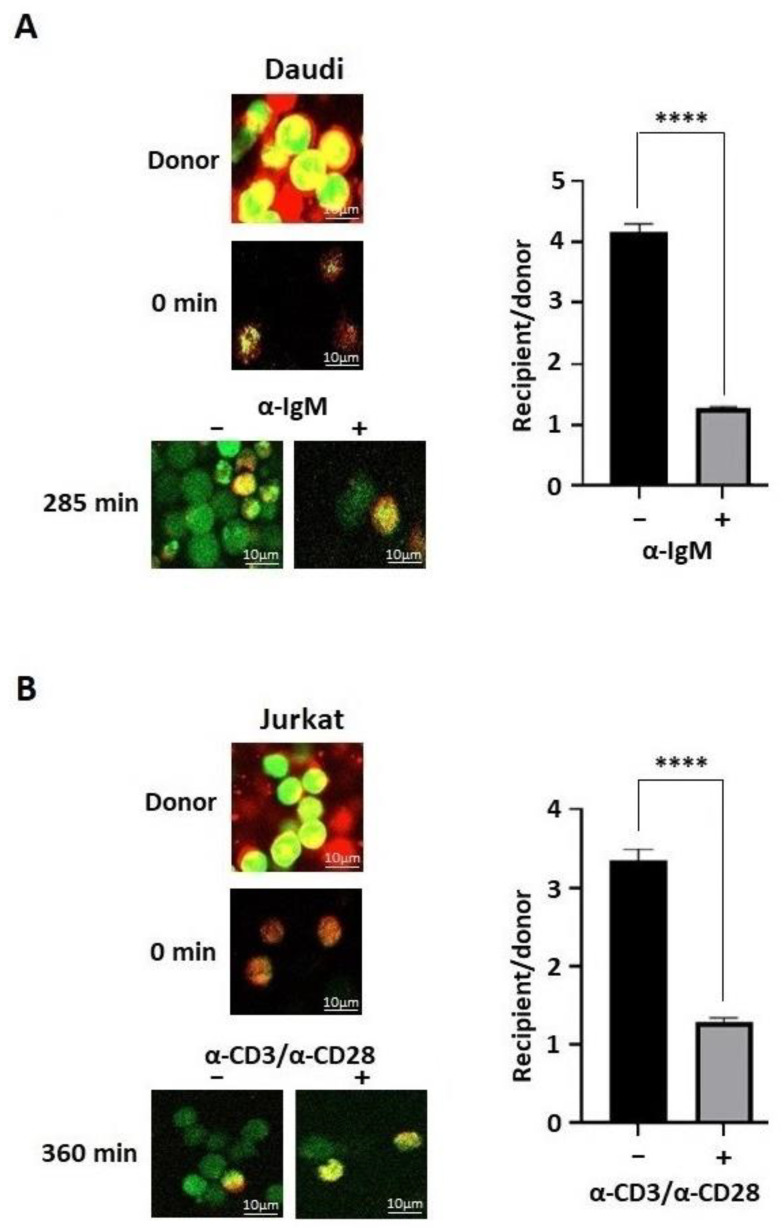
The effect of BTK and ITK phosphorylation on the Cx43 gap junction intercellular communication in lymphocytes. A parachute dye coupling assay was used to assess communication from the (**A**) Daudi cells with and without BTK activation using the α-IgM antibody or (**B**) Jurkat cells with and without ITK activation using the α-CD3/α-CD28 antibodies. Representative confocal images (Red, Dil; Green, calcein; Yellow, donor cells) are provided. Quantification was conducted by GraphPad Prism (units represent number of cells). Data represent the Student’s *t*-test (*n* = 3, **** *p* < 0.0001).

Because the lymphocytes had difficulty adhering to a surface to perform a scrape loading assay, a parachute assay was used to determine the effect of BTK and ITK activation on the Cx43 gap junction intercellular communication in the Daudi and Jurkat cells, respectively (Figure 9A,B). The donor cells loaded with Dil (red) and calcein AM, which converts into calcein (green), were incubated with the recipient cells and after the indicated times, the number of cells adjacent to the donor cell was counted. For both the Daudi and Jurkat cells, the activation of BTK and ITK by the B- (α-IgM) and T- (α-CD3/α-CD28) cell receptors led to a decrease in the number of adjacent cells taking up the calcein dye. The data were similar to those observed from the HEK-293T cells in that the cellular response to active BTK and ITK is to decrease the Cx43 mediated gap junction intercellular communication.

## 4. Discussion

When immune cells activate or are exposed to inflammatory factors, Cx43 gap junction channels transfer immuno-relevant signals such as ions, second messengers, small metabolites, microRNA, and peptides between neighboring cells [70]. Cx43 is the main gap junction protein of the immune system and localizes to an area between adjacent cells called the immunological synapse [52]. This specialized contact interface functions to prime and activate T and B cells by professional antigen presenting cells. This leads to the killing of infected cells by cytotoxic T lymphocytes and antigen extraction, processing, and presentation by B cells [71,72,73]. Several studies have attempted to deduce the importance of Cx43 at the immunological synapse, which include observations of direct cell-to-cell communication (T- and B-cell activation as well as antigen presentation), hemichannel activity (migration and autocrine/paracrine activation), and non-channel scaffolding functions [53,70,74,75,76].

T- and B-cell receptor signaling involves the activation of Akt, MAPKs, and PKC and an increase in intracellular Ca^2+^ and calmodulin activation [43,77]. Solan and Lampe (2016) put forth a spatiotemporal kinase model by which 5–15 min post injury or growth factor treatment, Akt phosphorylation of the Cx43 residue pS373 eliminates the Cx43 interaction with ZO-1 to accumulate Cx43 into larger gap junction plaques with increased intercellular communication. Next (15-30 min), Cx43 phosphorylation on S368 via PKC and on S255, S279, S282 via MAPK affect the gap junction channel gating properties and/or is associated with decreased gap junction assembly and increased gap junction turnover. Then (at 30 min extending for hours), Cx43 phosphorylation on Y247, Y265, and Y313 via Src inhibits microtubules and Drebrin (binds actin) binding to promote connexosome formation [36,43,77,78]. Of note, in a plasmacytoma B cell line, the phosphorylation of Cx43 residues Y247 or Y265 was also necessary for spreading [57]. This timeframe scale is consistent with kinase activation observed after T- and B-cell receptor activation [79,80]. In fact, B-cell receptor clustering in WEHI 231 immature murine B cells caused an increase in the slower migrating Cx43 isoform on SDS-PAGE gels caused by phosphorylation [81]. The change was first seen at 5 min, persisted for 15 min, and then started to decline at 30 min [81]. A point of consideration when comparing Cx43 data from different cell lines is the expression of their scaffolding proteins. For example, in the immune system, Drebrin is expressed in T lymphocytes and mast cells, but not in B lymphocytes. A future avenue of study is the potential for another Drebrin family member, the mammalian actin-binding protein 1 (mAbp-1) to play a role with Cx43 in B lymphocytes [78,82]. Additionally, there is no evidence to date that ZO-1 is found at the immunological synapse, unlike the other known family member to bind Cx43, ZO-2 [83]. To confirm these findings herein, we found no evidence of ZO-1 expression in the Daudi and Jurkat cells, and the expression of Drebrin was only in the Jurkat cells. These differences in Cx43 scaffolding partners may participate to alter Cx43 regulation suited for the immune system.

The significance of this study is that as part of the T- and B-cell receptor signaling, ITK and BTK were activated unlike Src, and we identified that both of these kinases affect Cx43 in a similar manner as Src. For example, ITK and BTK phosphorylate Cx43 residues Y247, Y265, and Y313, which led to a downregulation of the gap junction intercellular communication. This was observed in a kinase overexpressing system (HEK^BTK^ and HEK^ITK^) and when the kinases were receptor activated (Daudi, α-IgM and Jurkat, α-CD3/α-CD28). One difference observed between these cell lines was in the Cx43 migration pattern on an SDS-PAGE gel. In the Western blot from the HEK-293T cell lysate using the Cx43 antibody, the ITK and BTK phosphorylation of Cx43 caused the bands to collapse from the P2 (associated with gap junction intercellular communication) state to the P1 and P0 states [58,84]. Conversely, the expression level and pattern were relatively unchanged after activation of the T- and B-cell receptors. When using a Cx43 phospho-specific antibody, the level of pY247 increased in the presence of the kinases with the dominant isoforms being the P0 state in HEK^BTK^ cells and P1, P0, and P-1 in the HEK^ITK^ cells. A different result was observed when using the Daudi and Jurkat cells in that the activation of their T- and B-cell receptors, respectively, led to an increase in pY247, which was observed in hyperphosphorylated isoforms (P3 and P4). Our lab previously identified that the location of the tyrosine and serine phosphorylation sites (i.e., next to a proline or multiple next to each other) would cause these slower migrating isoforms [58]. Additionally, tyrosine phosphorylation at Y247, Y265, and Y313 alone together did not cause a change in the migration pattern [58]. This indicates that a significant level of serine phosphorylation also occurred on the carboxyl terminal domain after T- and B-cell receptor activation. This migration pattern was also observed in another more native like cellular system, hypertension human left ventricle myocytes, for which Cx43 stays membrane localized [24,85]. Interestingly, a study characterizing Cx43 phospho isoforms observed the presence of the P3 band when Cx43 redistributed from cell-to-cell interfaces to cytoplasmic locations in mitotic cells [86]. Cellular processes necessitating the disruption of a physical connection between cells would be cellular division, as well as is in mobile cells such as circulating erythrocytes and mature sperm cells, which do not express connexins. It would be tempting to speculate that the slower migrating isoforms observed after T- and B-cell activation were closed Cx43 channels remaining at the plasma membrane, thus maintaining the scaffolding functions. Consistent with this possibility is the fact that the cellular localization of Cx43 was relatively unchanged after T- and B-cell receptor activation. Another potential function of Cx43 remaining at the plasma membrane in a closed state is to help facilitate cell migration, but be prepared to aid in extravasation [70].

We have previously identified that Pyk2 and Tyk2 also phosphorylate Cx43 at residues Y247, Y265, and Y313 with a similar cellular fate to that of Src, ITK, and BTK [24,47,48]. Cx43 has a remarkably short half-life of only a few hours whether in cultured cells or native tissue [12]. There is still no definitive reason why such a rapid gap junction channel replacement is necessary, albeit the likely possibility is to respond quickly to physiological requirements to alter gap junction coupling. With the phosphorylation of Cx43 critical to the degradation of channels and kinase expression varying between different cell types, there is the need for different kinases to achieve the same function. The work presented herein points to ITK and BTK playing that role upon T- and B-cell activation.

## Figures and Tables

**Table 1:** Phospho-Tyr containing peptides identified from the mass spectroscopy of the Cx43CT in vitro phosphorylated by BTK.

Peptide Sequence	Start–End	Peptide Mass	Peptide Mass	Number of Phospho-Peptides	Phosphorylation Site
	(Residue Number)	(Calculated)	(Actual)		
SDPyHATTGPLSPSK	244–258	1557.6	1637.72	110	Y247
yAYYNGcSSPTAPLSPMSPPGYK	265–287	2435.7	2588.09	39	Y265
YAyFNGcSSPTAPLSPMSPPGYK	265–287	2435.7	2588.09	12	Y267
YAYFNGcSSPTAPLSPMSPPGyK	265–287	2435.7	2588.09	9	Y286
yAYFNGcSSPTAPLSPMSPPGyK	265–287	2435.7	2668.05	1	Y265/Y286
QASEQNWANySAEQNR	304–319	1895.9	1975.79	80	Y313

y-Phospho (+79.97 mass); c-Carbamidomethyl (+57.02 mass).

**Table 2:** Phospho-Tyr containing peptides identified from the mass spectroscopy of Cx43CT in vitro phosphorylated by ITK.

Peptide Sequence	Start–End	Peptide Mass	Peptide Mass	Number of Phospho-Peptides	Phosphorylation Site
	(Residue Number)	(Calculated)	(Actual)		
SDPyHATTGPLSPSK	244–258	1557.6	1637.72	50	Y247
yAYYNGcSSPTAPLSPMSPPGYK	265–287	2435.7	2588.09	33	Y265
YAyFNGcSSPTAPLSPMSPPGYK	265–287	2435.7	2588.09	16	Y267
YAYFNGcSSPTAPLSPMSPPGyK	265–287	2435.7	2588.09	24	Y286
yAyFNGcSSPTAPLSPMSPPGYK	265–287	2435.7	2588.09	5	Y265/Y267
yAYFNGcSSPTAPLSPMSPPGyK	265–287	2435.7	2668.05	16	Y265/Y286
QASEQNWANySAEQNR	304–319	1895.9	1975.79	80	Y313

y-Phospho (+79.97 mass); c-Carbamidomethyl (+57.02 mass).

## Data Availability

The data can be made available upon reasonable request to the authors.
